# HIV treatment outcomes among people who inject drugs in Victoria, Australia

**DOI:** 10.1186/s12879-014-0707-9

**Published:** 2014-12-19

**Authors:** Nick Walsh, Anne Mijch, Kerrie Watson, Handan Wand, Christopher K Fairley, John McNeil, Nick Crofts, Lisa Maher

**Affiliations:** Department of Epidemiology and Preventive Medicine, Monash University, 89 Commercial Road, Melbourne, 3004 Victoria Australia; Department of Medicine, Monash University, Clayton, 3065 Victoria Australia; Infection Prevention and Healthcare, Epidemiology Infectious Diseases and Microbiology Unit, Alfred Health, Melbourne, 3004 Victoria Australia; The Kirby Institute, Wallace Wurth Building, UNSW Australia, Sydney, 2052 NSW Australia; Melbourne Sexual Health Centre, Melbourne Sexual Health Centre, University of Melbourne, 580 Swanston St, Carlton, 3053 Victoria Australia; School of Public Health and Preventive Medicine, Monash University, 89 Commercial Road, Melbourne, 3004 Victoria Australia; Nossal Institute for Global Health, 4th Floor, 161 Barry St, Carlton, 3010 VIC Australia

**Keywords:** HIV, PWID, IDU, Mortality, Australia

## Abstract

**Background:**

People who inject drugs (PWID) are a key population affected by HIV. We assessed the effectiveness of HIV treatment among a clinical cohort of people living with HIV (PLHIV) diagnosed and referred for community-based antiretroviral therapy (ART) in Victoria, Australia.

**Methods:**

HIV notification data from a central statewide registry were matched with HIV clinical data from two large HIV treatment centers in Melbourne. We used survival analysis and Cox proportional hazard models to estimate time to AIDS and death for PWID in HIV treatment, compared with non-injectors, in the period 1996 – 2008.

**Results:**

Of the 871 individuals, 93 (10.8%) had injecting as an exposure category and 671 (86%) had ever commenced ART. Adjusted analysis showed younger age, high initial CD4 cell count (>500 cells/mm^3^) or ever having a CD4 cell count >500/mm^3^, and more recent calendar year of ART commencement were all associated with reduced hazards for AIDS and death, while older age, low initial CD4 cell count (<200/mm^3^), ever having a CD4 count <200/mm^3^ (before or during treatment) and high initial viral load (>5 log_10_) were associated with increased risk of AIDS and death. PWID were no more likely to experience AIDS (HR 0.98[0.54 – 1.80]) or death (HR 0.78 [0.18 – 3.42]) than non-injectors.

**Conclusion:**

Survival of HIV-infected PWID on HIV treatment was equivalent to non-injectors. CD4 cell count, initial viral load, calendar year of commencing ART and age are more important determinants of AIDS and mortality than injecting status for in-treatment PLHIV in Victoria, Australia.

**Electronic supplementary material:**

The online version of this article (doi:10.1186/s12879-014-0707-9) contains supplementary material, which is available to authorized users.

## Background

People who inject drugs (PWID) are a key population affected by HIV [1]. In Australia the prevalence of HIV among PWID has remained steady at around 1 – 2% since the mid 1980s [2]. Around 70% of HIV diagnoses in Australia are attributed to male-to-male sexual contact, while just over 9% are attributed to heterosexual sexual contact. Approximately 3% of HIV diagnoses in Australia are attributed solely to injecting drug use; this proportion has remained steady since the early 1990s [3],[4]. While the proportion of diagnoses attributable only to injecting drug use remains low, individuals are often in more than one risk population for HIV acquisition, such as men who have sex with men (MSM) *and* PWID. When including grouping for multiple exposure categories, the proportion of HIV diagnoses in Australia with injecting as an exposure category increases to almost 8% [3].

While the effectiveness of HIV treatment with antiretroviral therapy (ART) has improved substantially over the last 15 years, since the advent of highly active antiretroviral therapy (HAART) to the point where an HIV diagnosis may not markedly reduce life expectancy [5], PWID in many countries, especially low- and middle-income countries, continue to have poorer access to and outcomes from HIV treatment compared with their non-injecting peers, even when adherence is taken into account [6],[7]. Nevertheless, there is growing evidence that with appropriate support, PWID can achieve good clinical outcomes during HIV treatment [8]-[11].

To date, no studies have examined HIV treatment among PWID in Australia, a low HIV prevalence country with a State-supported universal health care system and an early and strong public health response to HIV [12]. The current study assesses the effectiveness of HIV treatment among a clinical cohort of people living with HIV (PLHIV) diagnosed and treated in Victoria, Australia, to determine the effectiveness of HIV treatment for PWID in the post-HAART era.

## Methods

HIV is a notifiable disease in Australia. In Victoria, HIV diagnoses are recorded in a central registry. Demographic and risk group data are recorded at diagnosis. This is periodically updated. Death in a person with HIV is also notifiable to the central HIV registry in addition to the civil registry of deaths.

We matched notification data of HIV diagnoses with clinical data from patients attending the Alfred Hospital HIV treatment clinic (the Victorian HIV/AIDS Service - VAHS) and the Melbourne Sexual Health Centre (MSHC) for HIV treatment. The vast majority of the approximately 4,000 PLHIV in Victoria live in Melbourne. These two sites were chosen as they treat a large proportion of PLHIV seeking treatment in Victoria. For example, our dataset contained 35% of all people diagnosed with HIV in Victoria between 1996 and 2005. As clinical data of interest was from the time period between 1996 and 2008 i.e. post-HAART, individuals were included where first contact with the treatment service (VHAS or MSHC) was between 1996 and 2008 and having had an HIV diagnosis prior to 2007. Records were matched using universal medical record numbers (where available) or name codes combined with date of birth and gender. Matching of the Victorian HIV Registry and the Alfred Hospital HIV treatment clinic generated 2,720 individual matches and eight duplicates. Matching of the Victorian HIV Registry and the Melbourne Sexual Health Centre HIV clinical data provided an additional 58 individuals. Merging these matched datasets resulted in clinical data being available for 2,868 individuals after records where individuals were aged < 13 were deleted from the dataset. A numerical individual identifier was generated for each case/patient and other identifying information removed.

**Figure 1:**
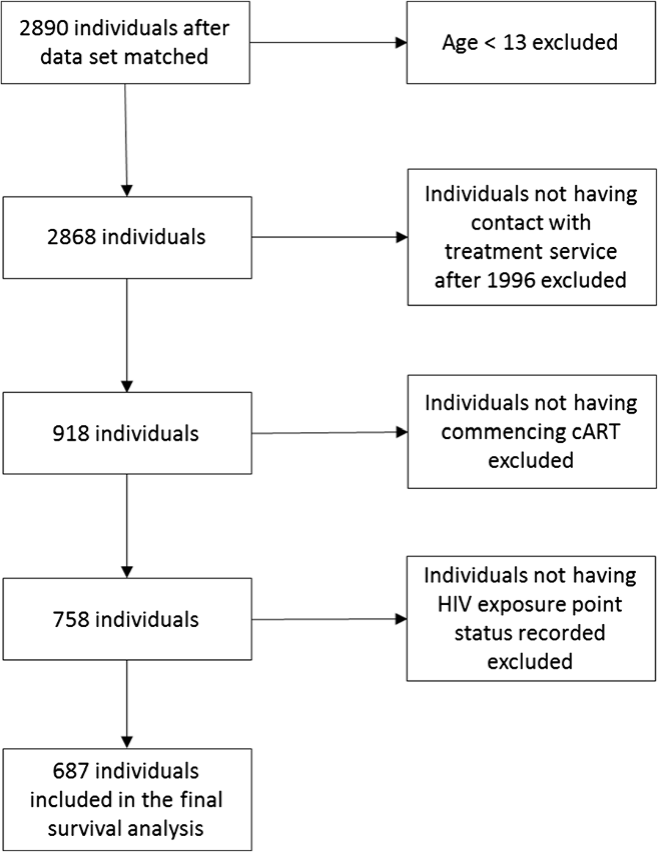
**Data management flow.**

Data on exposure category for HIV acquisition was taken from the Victorian HIV registry. Exposure categories included injecting, male-to-male sex, heterosexual sex or unknown. Where more than one exposure was present, both were recorded, e.g. an individual could have both injecting drug use and male-to-male sex as exposures for HIV. Mortality data was recorded in both the Alfred Hospital dataset and the Victorian HIV registry. Death was accepted if it occurred in either dataset. We examined completeness and found consistency between the datasets (data not reported). Data for AIDS diagnosis and other clinical data were recorded consistently in the Alfred and MSHC datasets and inconsistently in the Victorian HIV registry. Therefore, clinical data was only derived from the Alfred Hospital and MSHC datasets. Data management following matching and deduplication is shown in Figure 1.

### Variables of interest

Demographic characteristics included age, gender, indigenous status and country of birth. The single individual identified as transgender was included in the male category for the purposes of analysis. Risk group classification was derived from exposure category for HIV transmission at HIV diagnosis and categorized as PWID or non-injector, MSM or heterosexual, including belonging to more than one risk group. First recorded CD4 or viral load test result was referred to as ‘initial’ result. AIDS diagnosis was defined as date of first recorded AIDS defining illness. Mortality data were recorded.

### Analysis

Characteristics of the baseline study population were compared using the Pearson *x*^*2*^ test for dichotomous variables, the student’s t-test or Wilcoxon rank-sum test for continuous variables. Time points during HIV diagnosis were date of diagnosis, date of contact with the treatment service (either the VAHS or MSHC) and date of initiation of HIV treatment. Age at diagnosis, age at contact with the treatment service or age at commencement of HIV treatment were categorized into groups based on the distribution of ages in the dataset. CD4 cell count and viral load were dichotomized and categorized based on commonly used categories. For CD4, this was 0 – 199, 200 – 349, 350 – 499 and > 500 cells/mm^3^. For viral load, this was 0 – 3.99 log_10_, 4 – 4.99 log_10_ and ≥5 log_10_. In the survival analysis we examined only those individuals who ever commenced ART (n = 758, though only 687 had data on injecting status). Failure in the survival analysis was defined as AIDS defining illness diagnosis (first recorded) or death; otherwise records were censored at the last recorded contact with the treatment service. The time unit for all analyses was days from HIV diagnosis. The analysis for AIDS included 603 individuals, with a mean time at risk of 1,482 days (~3 years and 10 months). There were 108 failures (AIDS diagnoses). The analysis for death included 687 individuals, with a mean time at risk of 2,694 days (7 years 4 months and 15 days). There were 47 failures (deaths). Adjusted and unadjusted analyses were performed using death and AIDS separately. Variables achieving p < 0.1 in unadjusted analysis were included in Cox Proportional Hazards adjusted models, with the exception of PWID, given the a priori centrality to the hypothesis. For calendar year of initiation of HIV treatment, HIV diagnosis and first contact with the treatment service, years were grouped into three-year periods due to the low number of individuals ever having commenced HIV treatment.

Ethics approval for the study was provided by the Victorian Department of Human Services Human Research Ethics Committee and the Alfred Hospital Human Research Ethics Committee. Data were analyzed using Stata 11 (StataCorp, College Station, TX, USA).

## Results

Table 1 illustrates baseline characteristics of study participants stratified by history of injecting drug use. There were 918 individuals included in the final analysis, of which 871 had a recorded exposure category for HIV acquisition, including 628 (79.4%) MSM and 93 (10.8%) PWID. PWID were significantly younger than non-injectors, while the gender distribution was consistent between the groups. Females accounted for around 11% in both groups. There were no differences between PWID and non-injectors in initial CD4 cell count (362 vs 365.5 cells/mm^3^ p = 0.51) or initial viral load (27,150 vs 34,100 copies p = 0.79). There were also no differences in the median number of CD4 count tests or viral load tests between patients with a history of injecting drug use and those with no history. Of the 687 individuals who had ever commenced ART, 620 (90.2%) had no history of injecting drug use and 67 (9.8%) had a history of injecting drug use. Overall, AIDS and death were proportionally more common in non-injectors compared with PWID. Of those that had ever commenced ART, AIDS was more common in non-injectors, while there was no difference in the proportion of deaths comparing PWID with non-injectors.

**Table 1:** **Baseline data including demographics by injectin** **g status**

		Non injectors		PWID			
		No.	%	No.	%	Total	p
	**Total**	777	100	94	100	871	
**Exposure category**	**Heterosexual**	149	19.2	30	31.9	179	
	**MSM**	628	80.8	64	68.1	692	
**Gender**	**Male**	713	91.8	86	91.5	799	p = 0.001**
	**female**	63	8.1	8	8.5	71	
	**transgender**	1	0.1	0	0.0	1	
**Age at first recorded contact with treatment service**	**Median, IQR**	33.4		30.1			p < 0.001***
**Age (years)**	**<25**	37	4.8	11	11.7	48	
	**25 - 34**	242	31.1	43	45.7	285	
	**35+**	498	64.1	40	42.6	538	
**Initial CD4 cell count (cells/mm3)**	**Median number of CD4 records**	11		10			p = 0.192
	**median, IQR**	365.5		362			p = 0.461
	**<200**	222	29.2	15	16.3	237	p = 0.506
	**200 - 349**	130	17.1	27	29.3	157	
	**350 - 499**	150	19.7	18	19.6	168	
	**500+**	258	33.9	32	34.8	290	
	**Total**	760	100.0	92		852	
**Ever CD4 count < 200**		56	7.4	5	8.2	61	p = 0.497
**Ever CD4 count > 500**		506	66.6	62	10.9	568	p = 0.876
**Viral load**	**Median number of VL records**	12		10	3 – 19		p = 0.785
**Initial viral load (log**_**10**_ **copies)**	**Median IQR**	34100		27150	5700 – 74900		p = 0.759
	**<4 log**	263	34.7	31	10.5	294	
	**4 - 4.99 log**	261	34.4	44	14.4	305	
	**5+ log**	235	31.0	19	7.5	254	
	**Total**	759	100	94	11	853	
**Year of first recorded contact with treatment service**	**1996 - 1999**	305	39.3	31	9.2	336	
	**2000 - 2002**	236	30.4	30	11.3	266	
	**2003 - 2008**	236	30.4	33	12.3	269	
	**Total**	777	100	94	10.8	871	
**Ever started ART**		620	79.8	67	9.8	687	
**Year of Commencement of ART**	**1996 – 1999**	83	133.9	10	10.8	93	
	**2000 – 2002**	137	221.0	10	6.8	147	
	**2003 – 2005**	183	295.2	18	9.0	201	
	**2006 - 2008**	214	345.2	29	11.9	243	
	**Total**	620	100	67	9.8	687	
**Ever AIDS (all)**		216	27.8	16	6.9	232	p = 0.026*
**Ever AIDS (ever ART)**		206	33.2	14	6.4	220	p = 0.040*
**Death (all)**		52	6.7	4	7.1	56	p = 0.026*
**Death (ever ART)**		43	6.9	2	4.4	45	p = 0.214

* for p<.05, ** for p<.01, and *** for p<.001.

Table 2 shows adjusted and unadjusted Cox Proportional Hazard ratios from the survival analysis. Exposure category, including history of injecting drug use, was not associated with increased hazards for AIDS or death in survival analysis. Gender was similarly non-predictive. Older age at diagnosis was a significant predictor of both AIDS and death in those aged above 35 years. Reduced initial CD4 cell count was strongly associated with AIDS and death in adjusted analysis. Higher initial CD4 cell counts were associated with better outcomes, with the greatest benefit evident in patients with an initial CD4 cell count above 500 cells/mm^3^ (HR 0.19 for AIDS CI95 0.11-0.30, p = 0.000; HR 0.32 for death CI95 0.10 - 0.97, p = 0.044). Ever having recorded a CD4 cell count < 200 cells/mm^3^ (before or during treatment) was strongly associated with increased hazards for AIDS (HR 3.61 CI95 2.25 – 5.82, p < 0.001) and death (HR 9.00 CI95 4.04 – 20.02, p < 0.001), while ever having recorded a CD4 cell count > 500 cells/mm^3^ (before or during treatment) was strongly associated with reduced hazards for AIDS (HR 0.30 CI95 0.21 -0.41, p < 0.001) and death (HR 0.08 CI95 0.04 - 0.19, p = 0.000), independent of other covariates. In unadjusted analysis, having a high (>5 log_10_) initial viral load was associated with an increased hazard ratio for AIDS, and remained significant following adjustment for other variables (HR 1.79 CI95 1.18 – 2.68, p = 0.006). There was no relationship between initial viral load and hazards for death.

**Table 2:** **Hazard Ratios for AIDS and Death for those ever on ART (Adjusted** ^++^ **and Unadjusted)**

		AIDS						Death					
		Unadjusted			Adjusted			Unadjusted			Adjusted		
		HR	CI 95	P	HR	CI 95	P	HR	CI 95	P	HR	CI 95	P
**Risk group**	**Non injecting drug use**	Ref			Ref			Ref			Ref		
	**Injecting drug use**	0.70	0.39 -1.27	0.242	0.98	0.54 - 1.79	0.944	0.49	0.12 - 2.04	0.328	0.76	0.17 - 3.3	0.709
	**Heterosexual**	Ref			Ref			Ref			Ref		
	**MSM**	1.24	0.83 -1.86	0.299	1.07	0.7 - 1.63	0.750	1.81	0.71 - 4.59	0.215	1.28	0.48 - 3.42	0.625
**Age at diagnosis (years)**	**< 25**	Ref			Ref			Ref			Ref		
	**25 - 34**	1.04	0.68 – 1.58	0.872	1.81	1.04 - 3.15	0.035*	0.84	0.37 – 1.90	0.677	1.11	0.39 - 3.12	0.850
	**35+**	1.34	0.88 – 2.04	0.170	3.59	1.88 - 6.85	0.000***	1.14	0.59 – 2.53	0.737	3.34	0.93 - 12.02	0.065
**Age at contact with treatment service (years)**	**< 25**	Ref			Ref			Ref			Ref		
	**25 - 34**	0.87	0.47 – 1.58	0.639	0.46	0.21 - 0.98	0.045*	0.98	0.28 – 3.37	0.969	0.55	0.12 - 2.49	0.441
	**35+**	0.88	0.49 – 1.57	0.661	0.22	0.1 - 0.51	0.000***	0.91	0.28 – 3.02	0.881	0.22	0.04 - 1.17	0.075
**Initial CD4 count (cells/mm** ^**3**^ **)**	**<200**	Ref			Ref			Ref			Ref		
	**200 - 349**	0.18	0.11 -0.30	0.000***	0.18	0.11 - 0.3	0.000***	0.39	0.17 - 0.89	0.025*	0.54	0.22 - 1.3	0.167
	**350 - 499**	0.21	0.13 -0.34	0.000***	0.21	0.13 - 0.34	0.000***	0.31	0.12 - 0.80	0.015*	0.43	0.16 - 1.17	0.100
	**500+**	0.19	0.12 -0.29	0.000***	0.20	0.13 - 0.32	0.000***	0.17	0.06 - 0.49	0.001***	0.30	0.1 - 0.93	0.038*
**Ever CD4 (cells/mm** ^**3**^ **)**	**< 200** ^**§**^	3.06	2.05 -4.56	0.000***	3.61	2.25 – 5.82	0.000***	9.41	5.19 - 17.04	0.000***	9.00	4.04 – 20.02	0.000***
**Ever CD4 (cells/mm** ^**3**^ **)**	**> 500** ^**§**^	0.32	0.24 -0.43	0.000***	0.30	0.21 – 0.41	0.000***	0.09	0.04 - 0.19	0.000***	0.08	0.04 - 0.19	0.000***
**Initial viral load (log**_**10**_ **copies)**	**< 4 log**	Ref			Ref			Ref			Ref		
	**4 - 4.99 log**	1.23	0.83 -1.84	0.307	1.11	0.73 - 1.67	0.632	0.71	0.31 - 1.63	0.422	0.57	0.24 - 1.36	0.208
	**5 + log**	2.60	1.80 -3.77	0.000***	1.77	1.18 - 2.65	0.005**	1.86	0.94 - 3.67	0.075	1.76	0.81 - 3.85	0.154
**Year of diagnosis**	**< 1996**	Ref						Ref					
	**1996 - 1999**	1.45	1.00 -2.10	0.052				1.65	0.83 - 3.27	0.155			
	**2000 - 2002**	1.68	1.07 -2.65	0.025*				0.66	0.21 - 2.05	0.471			
	**2003 - 2005**	1.71	1.00 -2.94	0.050*				1.14	0.35 - 3.65	0.829			
**Year of commencement ART**	**< 1996**	Ref						Ref					
	**1996 – 1999**	0.53	0.16 -1.72	0.288	Ref		.	0.58	0.14 - 2.52	0.471	Ref		.
	**2000 - 2002**	0.30	0.09 -0.99	0.048*	0.58	0.35 - 0.97	0.036*	0.23	0.05 - 1.01	0.051	0.34	0.16 - 0.73	0.006**
	**2003 – 2005**	0.29	0.09 -0.92	0.036*	0.64	0.4 - 1.02	0.062	0.11	0.03 - 0.52	0.005**	0.19	0.08 - 0.42	0.000***
	**2006 - 2008**	0.31	0.10 -1.01	0.051	0.57	0.36 - 0.9	0.017*	0.01	0.00 - 0.09	0.000***	0.01	0 - 0.08	0.000***

* for p < .05, ** for p < .01, and *** for p < .001.

++In the adjusted analysis, non-significant covariates were dropped, apart from risk group. Gender, age of contact to the treatment clinic and age at ART commencement were excluded from the table as these were not significant in unadjusted analysis and so not included in adjusted analysis.

^§^ In adjusted analysis for ‘ever CD4 < or > 500’, initial CD4 count was excluded due to colinearity. The adjusted model for all other results here contains the variable ‘initial CD4 count’ rather than ‘ever CD4 < 200 or > 500’.

Calendar year of ART commencement was a key determinant of outcome. The relationship between calendar year of ART commencement and AIDS showed reduced hazards (compared with 1996 – 1999) from 2003 onwards, while for death the benefit was seen from 2000. While in unadjusted analysis the risk of AIDS was increased in those diagnosed with HIV in later years (2000 – 2005), after adjustment this was not significant. There was no relationship between calendar year of diagnosis and AIDS or death in adjusted analysis.

**Figure 2:**
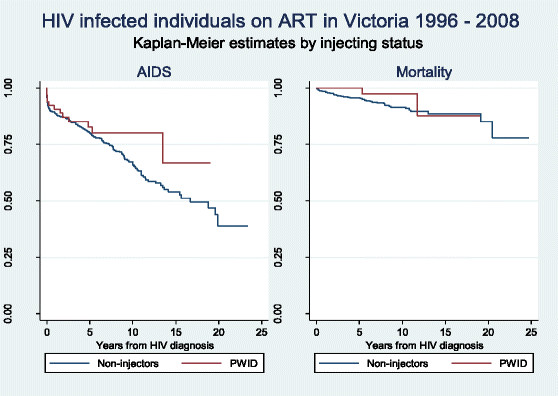
**Kaplan-Meier estimates for AIDS and Death by injecting status in Victoria 1996–2008.**

Figure 2 compares AIDS and mortality for the follow-up period 1996 – 2008 between patients with and without a history of injecting drug use. The Kaplan-Meier estimates were adjusted for age, year group of diagnosis and initial CD4 cell count shown to be important covariates in Table 2. As discussed above, injecting status did not influence hazard ratios for AIDS or death. In addition, an analysis was made of PWID only (i.e. heterosexual PWID), which also found no statistically significant difference in AIDS or death likelihood compared with the rest of the cohort (data not shown).

## Discussion

We found no difference in clinical outcomes for HIV treatment between patients with and without a history of injecting drug use in a real-world clinical cohort comprising the two largest HIV treatment services in Victoria, Australia. A number of large studies have examined HIV treatment among PWID in the post-HAART era. Several large collaborative cohort studies, as well as a number of smaller cohorts, have found that PWID have benefited less from HAART and continue to progress to AIDS and death at a faster rate than other risk categories [6],[7],[13],[14].

However, our results are consistent with several previous studies [11],[15]-[17]. The HERO and ALIVE cohorts from the east coast of the United States found that injecting drug use, and its treatment, were not associated with poorer HIV treatment outcomes compared with non-injectors among those with higher CD4 counts [15],[16]. In the HERO cohort, commencing HAART at CD4 count of 200 – 350 and > 350 was associated with improved survival compared with lower CD4 counts, whereas in an analysis of the ALIVE cohort of PWID, participants with CD4 counts below 200 fared significantly worse than those with counts above 500. The importance of CD4 cell count in HIV treatment is well documented [14]. In our study, the median initial CD4 count did not differ by injecting status and was reasonably high overall (just over 360); higher CD4 counts were associated with a reduced risk of AIDS and death, regardless of injecting drug use status. Achieving a CD4 count of over 500 at one point in time conferred a substantial decrease in AIDS or death, while ever having a CD4 count lower than 200 substantially raised the risk of developing AIDS, irrespective of injecting drug use status. A recent collaborative cohort study demonstrated that achieving a CD4 count above 500 was associated with a mortality rate similar to that of non-HIV infected populations, and while PWID did not benefit to the same extent, longer duration of CD4 count above 500 was associated with improved outcomes, even in PWID. Notwithstanding this latter finding, our study and others demonstrate the importance of achieving high CD4 counts in HIV treatment to reduce HIV-related mortality and morbidity [18].

A Spanish study of HIV-infected PWID in a tertiary hospital setting examined both pre and post-HAART era HIV survival and found a marked increase in survival in the post-HAART era, equivalent to non-HIV infected PWID [17]. This study was conducted in a substance use treatment setting including opioid substitution therapy (OST) which has been shown to improve adherence to, and the effectiveness of, HAART [19],[20]. A prospective observational cohort study in British Columbia also found no difference in mortality rates between HIV-positive PWID and other groups initiating HAART [11].

Our finding of improved outcomes by calendar year is consistent with other studies [7],[17]. There are a number of potential reasons for improved HIV treatment outcomes by calendar year, including a reduction in virological failure [21] and a shortened duration of HIV infection compared to individuals having entered our clinical cohort earlier.

There are a number of limitations to our study. Given the epidemiology of HIV in Australia, our sample of patients with a history of injecting drug use was small and the absolute number of deaths in this group was also small. Our dataset only included individuals who accessed services at two urban clinics, and we did not have access to treatment data from other sites. It has been reported that some HIV-positive PWID in Melbourne may be reluctant to access HIV therapy [22]. Others have reported that PWID residing near drug using areas are less likely to access HIV treatment even when it is available [23]. We did not have reliable data on cause of death, and as others have demonstrated, deaths in HIV disease are not always due to HIV infection [15]. Nevertheless, we would expect substance use deaths to be higher in the PWID group, potentially leading to an increased mortality rate in this group – which was not the case. In addition, the study was not able to assess loss to follow-up, so censoring occurred at the last reported clinical variable or study endpoint.

We used exposure category at HIV diagnosis as a population HIV risk classifier, whether or not it reflected ongoing drug use. We used this proxy as ongoing drug use data was not available. Although many studies also use risk category at diagnosis to define an individual’s at-risk population group [6],[7],[14]-[16], this may not always reflect impact of risk behavior. Potentially, this may have overestimated the number of PWID, though in which direction this may have pushed results is contentious.

This study was an analysis of clinical outcomes in a real-world setting, rather than a stand-alone cohort study. Health care in Australia operates under a universal Government-subsidized model, where patients are given care at no charge at public hospitals and State-funded primary health centers (such as the Alfred Hospital or Melbourne Sexual Health Centre). Elsewhere in the community, most general practice consultations are subsidized by the Federal Government, with people not on a low income or not receiving social welfare payments are subject to a small co-payment. Medication is provided through the National Pharmaceutical Benefits Scheme which provides Government subsidies for medication such that an individual pays a modest, maximum amount, less if they are receiving social welfare benefits or are on a pension. These factors minimize the impact of access to health care issues, despite our study being done in a real-world context. In addition to universal health care, adherence to HIV treatment guidelines by HIV clinicians in Australia has been documented as high [24]. As a consequence, we believe there is little cost and treatment bias influencing the outcomes of this study. While our study also compared patients with a history of injecting drug use and those without, similar to the Canadian study [11], HIV prevalence among PWID in Victoria is substantially lower than in Vancouver/British Columbia. It is not known if or how this may have impacted on our results.

## Conclusion

Our results indicate that in Victoria, patients with a history of injecting drug use benefit equally well from HIV treatment as those without a history of injecting drug use, including MSM and those acquiring HIV from heterosexual sexual transmission. In our study, the main determinants of HIV treatment outcomes remain CD4 cell count, viral load, age and calendar year of HIV diagnosis.

## Authors’ original submitted files for images

Below are the links to the authors’ original submitted files for images. Authors’ original file for figure 1 Authors’ original file for figure 2
