# Supplementary figures and images for: HIV treatment outcomes among people who inject drugs in Victoria, Australia
Source: BMC Infect Dis. 2014 Dec 19;14:707. doi: 10.1186/s12879-014-0707-9 (PMC4298908; doi:10.1186/s12879-014-0707-9)

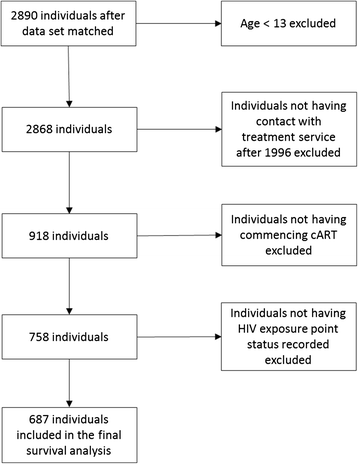

Supplement: Supplementary file 1 — Authors’ original file for figure 1 [file 12879_2014_707_MOESM1_ESM.gif]

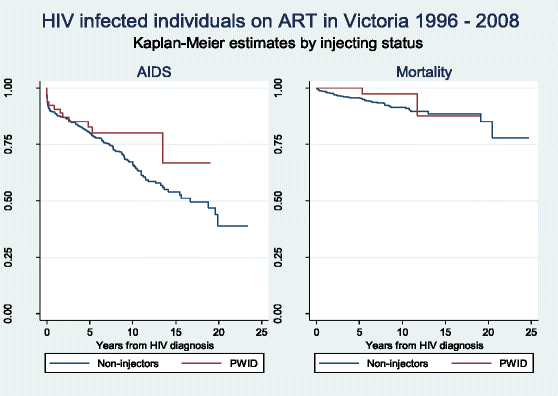

Supplement: Supplementary file 2 — Authors’ original file for figure 2 [file 12879_2014_707_MOESM2_ESM.gif]
